# Characterization of Nile Red as a Tracer for Laser-Induced Fluorescence Spectroscopy of Gasoline and Kerosene and Their Mixture with Biofuels

**DOI:** 10.3390/s19122822

**Published:** 2019-06-24

**Authors:** Matthias Koegl, Christopher Mull, Kevin Baderschneider, Jan Wislicenus, Stefan Will, Lars Zigan

**Affiliations:** 1 Lehrstuhl für Technische Thermodynamik (LTT), Friedrich-Alexander-Universität Erlangen-Nürnberg (FAU), 91058 Erlangen, Germany; christopher.mull@fau.de (C.M.); kevin.baderschneider@fau.de (K.B.); jan.wislicenus@fau.de (J.W.); stefan.will@fau.de (S.W.); lars.zigan@fau.de (L.Z.); 2 Erlangen Graduate School in Advanced Optical Technologies (SAOT), Friedrich-Alexander-Universität Erlangen-Nürnberg (FAU), 91052 Erlangen, Germany

**Keywords:** gasoline, Jet A-1, dye, nile red, absorption, ethanol

## Abstract

Suitable fluorescence tracers (“dyes”) are needed for the planar measurement of droplet sizes by using a combination of laser-induced fluorescence (LIF) and Mie scattering. Currently, no suitable tracers have been characterized for application in planar droplet sizing in gasoline and kerosene fuels, as well as biofuel blends. One promising tracer is nile red, which belongs to the fluorophore group. For its utilization for droplet size measurements, preliminary characterization of the fluorescence of the respective fuel tracer mixtures are mandatory. For this purpose, the fluorescence and absorption behavior of nile red dissolved in the surrogate fuels Toliso and Jet A-1 as well as in biofuel blends was investigated. The fluorescence signal for nile red that was dissolved in the two base fuels Toliso and Jet A-1 showed a linear behavior as a function of dye concentration. The temperature effect on spectral absorption and emission of nile red was investigated in a specially designed test cell. An ethanol admixture to Toliso led to a spectral shift towards higher wavelengths. The absorption and emission bands were shifted towards lower wavelengths with increasing temperature for all fuels. Both absorption and fluorescence decreased with increasing temperature for all fuels, except for E20, which showed an increased fluorescence signal with increasing temperature. Jet A-1 and its blends with hydroprocessed esters and fatty acids (HEFA) and farnesane did not exhibit explicit variations in spectral absorption or emission, but these blends showed a more distinct temperature dependence compared to the Toliso-ethanol-blends. The effect of photo-dissociation of the LIF signal of the fuel tracer mixtures was studied, and all fuel mixtures besides Toliso showed a more or less distinct decay in the fluorescence signal with time. In summary, all investigated fuel-tracer mixtures are suitable for LIF/Mie ratio droplet sizing in combination with nile red at moderate temperatures and low evaporation cooling rates.

## 1. Introduction

Liquid fuel atomization and evaporation behavior controls the subsequent processes of ignition, heat release, and pollutant formation, as well as the efficiency of a combustor. This process chain is complex and not fully understood, yet the utilization of alternative fuels introduces an even higher degree of complexity to the combustion process. 

In the automotive sector, gasoline-ethanol blends are nowadays widely used. The time for atomization and mixture formation of modern direct injection spark-ignited (DISI) engines is very short, and the combustion process is very complex. An admixture of ethanol to commercial gasoline leads, under certain conditions, to an increase of soot formation within a DISI-engine [1]. An admixture of 20 vol% ethanol (E20) showed an enhanced soot formation in comparison to the base fuel and E40 (60 vol% gasoline, 40 vol% ethanol). Here, the thermo-physical fuel properties play a major role for the atomization and mixture formation, which governs, for example, the soot formation [2]. The usage of biofuel components in automotive and aviation applications can lead to an essential contribution for reaching future CO_2_-emission limits. 

In aviation applications, the investigation on the emission of bio-components at research flights and its usage in civil aviation has slowly begun. In 2014, a new biofuel called farnesane (2,6,10-trimethyldodecane), which has a similar chemical structure as common Jet A-1 (kerosene), received the admission of the ASTM (American Society for Testing and Materials) International for admixture to Jet-A1. Currently, the fuel is extracted from sugar and is thus CO_2_-neutral. Furthermore, it is free from the contaminants usually found in Jet A-1, a fuel that increases pollutant emissions in general [3]. Additionally, an admixture of 30 vol% of farnesane to fossil diesel fuel led to a significant reduction in CO and HC (hydrocarbon) emissions within a small diesel engine [4].

Recently, NASA and DLR investigated the particle formation of Jet-A1 blended with HEFA (hydroprocessed esters and fatty acids) [5], which are compounds extracted from plant oil and consists of pure paraffins without any aromatic hydrocarbons. Similar to IC (Internal Combustion) engines, the different chemical and physical properties of the biofuel components can significantly change the evaporation and atomization behavior of the base fuel in a gas turbine, and this has a high impact on the pollutant formation, especially the soot emission. In ref. [5], the impact of a biofuel admixture of 30% to ­50% on the soot emissions of a turbine was investigated. An admixture of 50% HEFA showed a reduction of particulate matter in the range of 50% to 70% in comparison to conventional Jet A-1. 

The investigation and optimization of the fuel-dependent spray formation is necessary, and this requires the application of optical techniques. Many optical techniques for spray analysis were developed during recent years [6]. Laser-induced fluorescence (LIF) constitutes a tool with a wide applicability for spray analysis. One approach is two-color-thermometry, where the temperature dependence of the fluorescence signal of organic dyes is used to determine the temperature in the liquid-phase (e.g., liquid solutions, individual droplets, and sprays) [7,8,9,10,11,12,13,14]. The two-line excitation laser-induced fluorescence technique enables the determination of the temperature within the vapor phase [15,16]. Temperature in the liquid and gas phases can also be determined with Raman spectroscopy [17,18,19,20,21]. Beyond that, LIF can also be used to determine the fuel/air-ratio within automotive sprays [15,22]. The combination of LIF and Mie-scattering, the so-called LIF/Mie ratio approach, yields droplet sizes in terms of the SMD (Sauter mean diameter). The determination of the droplet size distribution is also possible using the Raman/Mie ratio [23], the laser-induced exciplex fluorescence (LIEF)/Mie ratio [24], and MDR (morphology dependent resonances, or “lasing”) images of micro droplets [25]. The present study deals with the characterization of a tracer concerning its applicability for LIF/Mie droplet sizing, so the technique is described in more detail in the following section. A quantification of the droplet size distribution in terms of the SMD is possible with an adequate calibration, e.g., by using PDA (phase Doppler anemometry) measurements [2] or a droplet generator [26,27]. With a laser sheet scanning technique, averaged 3-dimensional quantitative droplet size distributions can be extracted with moderate effort [28], which is also relevant for the validation of spray models in computational fluid dynamics (CFD) simulations. 

The LIF/Mie ratio technique is based on the *d³*-dependence of the LIF-signal and the *d²*-dependence of the Mie-signal of laser-illuminated liquid droplets. The LIF-signal in a fuel spray is usually created by a tracer dissolved in a surrogate fuel [2,29] or by the aromatic components of the multi-component fuel itself [27]. While the Mie-signal is roughly temperature insensitive, the LIF-signal could be strongly temperature sensitive. The LIF-signal also depends on the solvent and the absorption and emission properties of the tracer at the respective conditions. In particular, the ambient temperature around a droplet may exert a strong influence on the fluorescence signal, which must be considered in the measurement uncertainty analysis. For example, the fuel droplets heat up in the hot ambience until they reach a quasi-steady temperature due to the simultaneous evaporation cooling during evaporation. During evaporation, the liquid and gas phase temperature within a spray can easily change by more than 10 K even under low and moderate ambient conditions (e.g., at 293 K) due to the evaporation enthalpy of the fuel [2]. Consequently, a temperature-insensitive fuel-dye-system has to be utilized in order to get reliable results.

Automotive and aviation fuels consist of a high amount of aromatic hydrocarbons that typically absorb laser light in the UV wavelength range. Consequently, when adding dyes for studying liquid fuel distribution or droplet sizes, these tracer substances should absorb laser light preferably in the visible wavelength range to ensure that only the tracer and not the fuel components gets excited by the laser.

Many studies on droplet sizing in alcohol or water sprays already exist in the literature [2,10,26,28,29,30,31]. Within these studies, the tracer eosin Y, which is almost temperature-insensitive at moderate ambient conditions, was frequently used [2]. A temperature-sensitive tracer like fluorescein is not suitable for the LIF/Mie droplet sizing approach [10]. Furthermore, eosin Y and fluorescein are not soluble in alkanes or gasoline [32].

However, few tracers are available for exploiting the fluorescence behavior in alkanes, oils, and commercial fuels. The dye pyrromethene (of which there are different derivatives, e.g. 597-8C9, 597-C8) is soluble in these fuels. However, due to its temperature sensitivity, it is not applicable for LIF/Mie droplet sizing but is commonly used for thermometry [33,34]. The tracer nile red (C_20_H_18_N_2_O_2_), which is mainly used in microfluidic systems and in biology applications [35,36,37], is another dye that can also be dissolved in real-world fuels [32]. However, its fluorescence characteristics have no yet been quantified for varying conditions, which is the topic of the present study. Furthermore, the absorption and emission behavior of the dye depends on the solvent. This is especially relevant for fuel mixtures containing biofuels, which may significantly affect the absorption and emission behavior. For example, in a mixture formation study using the tracer trimethylamine (TEA) dissolved in isooctane, both a larger red-shift of the fluorescence spectrum and a very low signal strength were observed with increasing ethanol content [22]. Koegl et al. [2] used the tracer eosin Y in ethanol and butanol and found different fluorescence signal strengths. This effect also showed up in a study by Chakraborty and Panda [38], who reported a higher fluorescence intensity of eosin Y in ethanol over the whole emission range compared to the solution with butanol. 

This article reports on fluorescence and absorption measurements of the dye nile red in a gasoline surrogate and in aviation fuels blended with bio-components, which was conducted to evaluate its applicability for LIF/Mie droplet sizing. For the investigations, the gasoline surrogate fuel “Toliso”, consisting of isooctane and toluene (65 vol%/35 vol%; see also Section 3 for further explanation) [1,39,40,41] mixed with ethanol (20 vol% in E20 and 40 vol% in E40), as well as four aviation fuel mixtures were analyzed. The aviation fuels studied are the base fuel Jet A-1 and blends with HEFA (30 vol% and 50 vol%) and with farnesane (10 vol%), which are approved by the ASTM for aviation applications. First, the influence of dye concentration on the fluorescence of the two base fuels Toliso and Jet A-1 was investigated. Afterwards, the individual spectra of the fluorescence and absorption of the dye dissolved in the different biofuel blends were analyzed for a wide range of temperatures in a specially designed test cell. Finally, a possible photo-dissociation effect was investigated, and the suitability of the application of this dye for LIF/Mie droplet sizing and further future work is discussed.

## 2. Description of the Experiment

### Experimental setup

**Figure 1:**
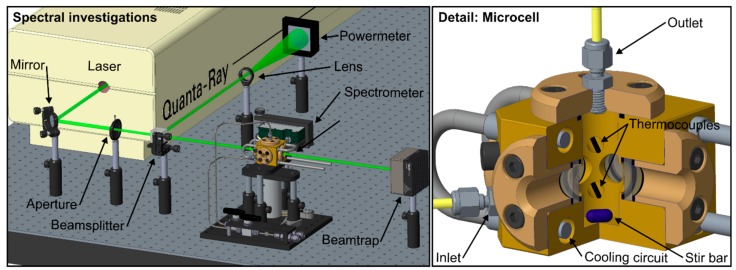
Optical arrangement of the laser-induced fluorescence (LIF)-setup (left); detail of the internal design of the microcell (right).

The optical arrangement of the LIF-setup and a detailed view of the internal construction of the microcell is shown in Figure 1. A Nd:YAG laser (model 150-10, Quanta Physics, USA) with a wavelength of 532 nm and a pulse width of <10 ns was used. An electric actuated shutter inside the laser enables continuous laser operation and a time-limited illumination of the probe volume during the measurements in order to prevent photo-dissociation effects. An aperture cuts the laser beam down to a diameter of 4.2 mm. Afterwards, a 50/50 beam-splitter separates the beam, and a power meter (model QE50LP-S-MB-INT-D0, Gentec Electro-Optics, Canada) was used for monitoring the laser fluence (which was adjusted to 45.8 mJ/cm²) during the measurements. After this, the beam passes the measurement volume in a specially designed cell (see below) or a cuvette (not shown), respectively. The LIF-spectra were recorded under a detection angle of 90° by a spectrometer (model USB 4000, Ocean Optics, USA, wavelength range 495.9–831.8 nm, 3648 pixels, slit size 10 µm, integration time 100 m, 50 subsequent spectra were averaged for each measurement). In general, the standard deviations of the fluorescence measurements were always below 0.5%. For studying the effect of photo-dissociation (or “photo-bleaching”, which was conducted at ambient conditions [0.1 MPa, 293 K]) on the fluorescence signal, a cuvette with quadratic cross section (edge length 10 mm) and a small volume (3.5 mL) was used to promote fast dye dissociation. A microcell was designed for the detection of temperature-dependent emission and absorption spectra. The microcell features four single ½" sapphire windows with an optical access diameter of 9 mm. The inner distance between the windows of the cell is 19.1 mm. To ensure a homogeneous temperature distribution within the cell, a magnetic stirrer (8 mm × 3 mm) was used in combination with two thermocouples (type K). A recirculating thermostat (type: Julabo FP50) was employed to enable a wide range of investigated temperatures. The temperature-dependent absorption measurements were performed using an UV/VIS spectrometer (model V-750, JASCO, Japan, wavelength range 190–900 nm, 3551 pixels, spectral bandwidth 2 nm, scan speed 200 nm/min; three subsequent spectra were averaged for each measurement) containing the microcell mounted inside. The baseline was determined by the absorption measurement of the microcell itself containing no fuel/dye solution.

## 3. Fuels and Dye Used

Gasoline contains up to 35 vol% of aromatic components such as toluene, benzene, or xylene, and these strongly determine the sooting tendency. Toluene, especially, is relevant as it acts as an octane number enhancer in gasoline with concentrations of up to 15 vol% [42]. In accordance with previous studies, we utilized a well-defined two-component surrogate fuel instead of gasoline. This surrogate fuel “Toliso” consists of 65 vol% isooctane and 35 vol% toluene. Toliso has similar thermo-physical properties as multi-component gasoline and has already been used in earlier studies addressing spray and mixture formation as well as sooting combustion [1,39,40,41]. Toluene is an aromatic hydrocarbon and, in mixtures with isooctane and n-heptane, it can achieve a similar sooting behavior as commercial gasoline [43]. Furthermore, the effects of biofuels were addressed so that Toliso was blended with 20 vol% or 40 vol% ethanol (E20 and E40). In the case of aviation fuels, the base fuel Jet A-1 was blended with typical fractions of biofuel (see above), namely 30 vol% or 50 vol% HEFA (Jet A-1 H30 and Jet A-1 H50, respectively) or 10 vol% farnesane (Jet A-1 F10) in order to study their effects on the fluorescence properties of nile red. In principle, kerosene could also be substituted by toluene and alkanes, see, e.g., Chen et al. [44], who used toluene and n-decane. However, no distinct variation of the absorption and emission behavior of this 2-component mixture compared to “Toliso” was expected. The chemical and physical properties of the investigated fuels are shown in Table 1.

**Figure 2:**
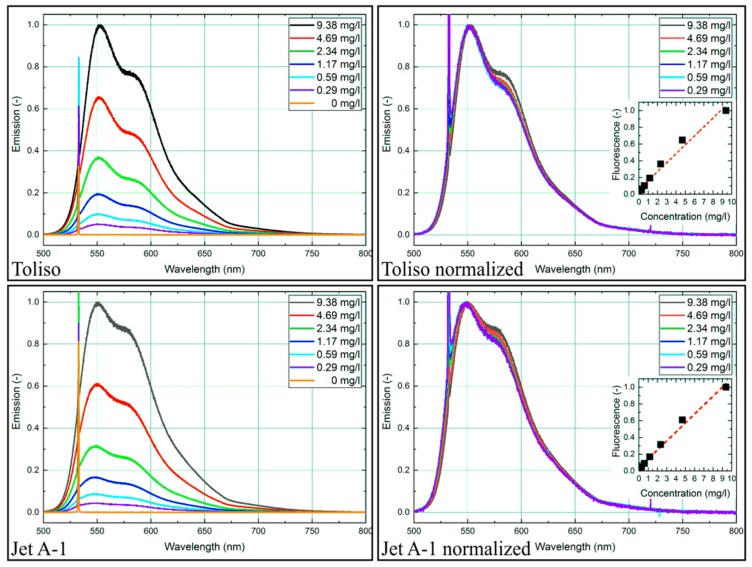
Emission spectra of nile red (left: normalized to maximum intensity at 9.38 mg/L; right: all spectra normalized to their respective maximum value; inserted diagram showing linearity of the integral LIF-signal) for various dye concentrations in Toliso and Jet A-1, 293 K, 0.1 MPa

The dye nile red (Sigma Aldrich) belongs to the fluorophores group, and its aromatic ring structure contains polar substituents. This fact leads to its sensitivity to the chemical and physical environment of surrounding solvent molecules [57]. Due to its high absorption cross-section, a low dye concentration of 9.38 mg/L (see Figure 2) was utilized unless otherwise noted. For the solutions, a minimum of 5 mg of nile red was weighed with an analytical scale (Mettler Toledo XS 205; proofed repeatability 0.05 mg). The tracer was completely dissolved in the fuel mixtures for all investigated concentrations.

## 4. Results

This section is structured as follows: First, the investigation on the effects of dye concentration on the fluorescence spectra of the two base fuels Toliso and Jet A-1 is presented. Second, the analysis of the temperature-dependent absorption and emission spectra of the investigated fuel-tracer-mixtures is reported. Third, the investigation on the effect of the solvent on the absorption and emission spectra is presented. Fourth, the effect of photo-dissociation for the various fuel-dye mixtures is shown.

### 4.1. Effect of Dye Concentration on Fluorescence Spectra

The concentration-dependent emission spectra of the two base fuels Toliso and Jet A-1 with nile red at reference conditions (293 K, 0.1 MPa) are shown in Figure 2. In both base fuels, nile red shows an emission between about 500 nm and 750 nm. A double-peak in the emission spectrum is visible; the left peak is more pronounced than the right peak, especially for Toliso. The emission intensities for both fuels decrease linearly with lower dye concentrations. The coefficient of determination R² for the linear fitting curves displayed in Figure 2 is 0.971 for Toliso and 0.986 for Jet-A1. The normalized graphs show a slight increase of the right peak for increasing dye concentrations. 

### 4.2. Temperature-Dependent Absorption and Emission Spectra

**Figure 3:**
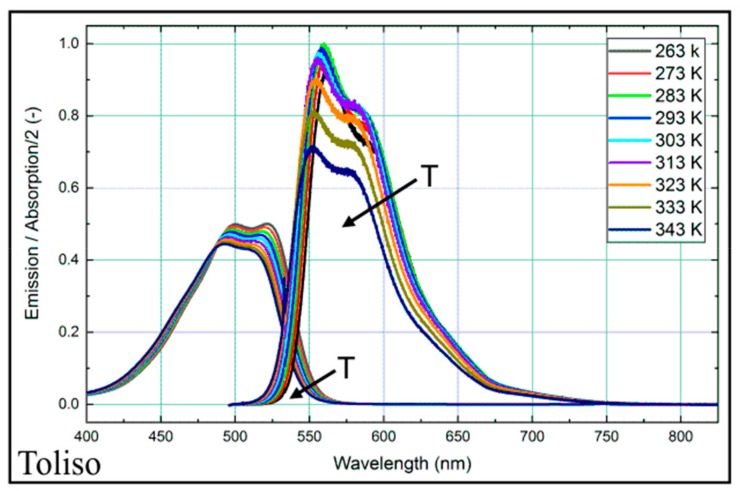
Normalized absorption (left curves) and emission spectra (right) for nile red (9.38 mg/L) in Toliso at various temperatures, 0.1 MPa. (Please note that the absorption signal is divided by a factor of two for clarity).

**Figure 4:**
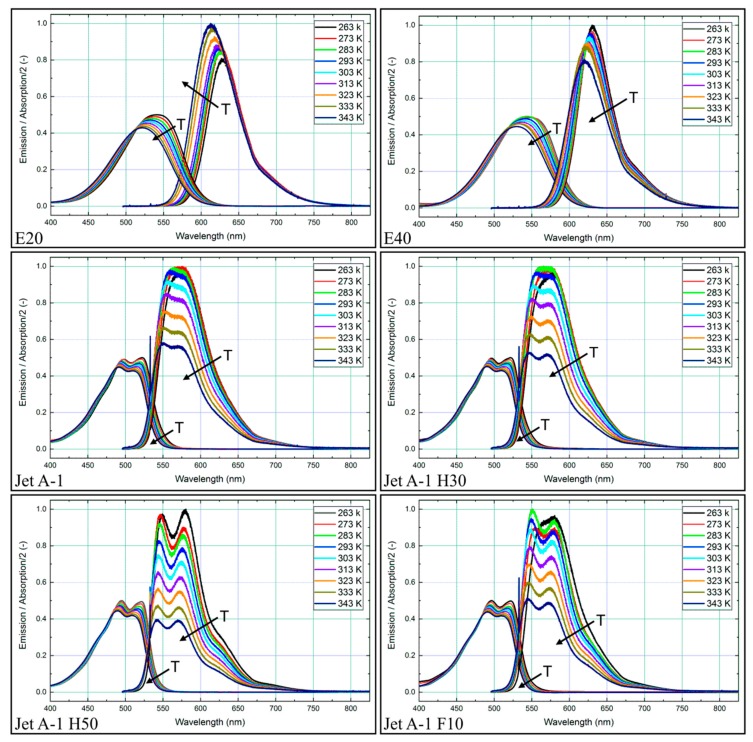
Spectral absorption and emission spectra for nile red (9.38 mg/L) in various fuels at various temperatures, 0.1 MPa. (Please note that the absorption signal is divided by a factor of two for clarity).

**Figure 5:**
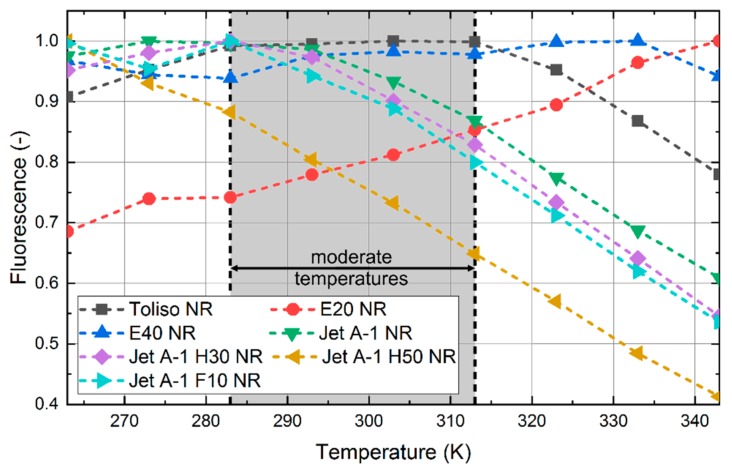
Normalized integral fluorescence intensities of the dye nile red in the investigated fuels for various temperatures, 0.1 MPa. All standard deviations are <0.5% and are smaller than the symbols. The moderate temperature domain is marked grey.

The temperature-dependent absorption and emission spectra for the Toliso-nile red-mixture are shown in Figure 3 (and for the other fuels in Figure 4). The absorption and emission spectra are presented for the visible wavelength region (400–750 nm), which is most relevant for the absorption and emission of nile red. For Toliso, below 400 nm, there are also absorption bands of toluene (240–270 nm) [58] and kerosene (245–330 nm) [59], which cannot be excited at 532 nm. The absorption and fluorescence signal mainly consists of continuous spectra without distinct structures except for two individual peaks. At lower temperatures, the integral fluorescence signal shows a weak temperature dependence in the temperature range of 283–323 K (less than 10%, see also Figure 5). At elevated temperatures, the signal intensity decreases by 22% between 313 K and 343 K, which is correlated to the absorption behavior. The absorption signal decreases constantly with higher temperatures (about 4% between 313 K and 343 K within 375–900 nm), which partly explains the decrease of fluorescence signal. Obviously, the fluorescence yield depends on temperature as well. The fluorescence and the absorption spectra are slightly shifted to lower wavelengths with increasing temperature. With higher temperatures, the first peak of the emission spectrum around 555 nm decreases more in comparison to the second peak at around 580 nm.

The absorption and emission spectra of the other fuel-nile red-mixtures are shown Figure 4. Here, the dependence of the fluorescence signal on the solvent becomes visible, which has already been reported by Kalathimekkad et al. [57] for the solvents toluene, methanol, ethanol, terpineol, acetone, and isopropyl alcohol. An ethanol admixture to Toliso leads to a spectral shift of the peaks of about 25 nm for the absorption and 50 nm for the fluorescence signal towards larger wavelengths. Instead of two peaks in the absorption and emission spectra, only one single peak remains. Again, all absorption and emission spectra are slightly shifted towards lower wavelengths with increasing temperature. With the exception of E20, which shows an increase of the (peak) intensity with higher temperatures, all other fuel-tracer mixtures show a decreasing intensity with increasing temperature. 

Nile red dissolved in Jet A-1, and its blends show a more distinct temperature dependence compared to the Toliso-blends. The blending of Jet A-1 with HEFA and farnesane leads to an increased temperature dependence of the fluorescence of nile red, while the highest temperature dependence occurs for Jet A-1 H50 with a decrease in intensity of 58% between 273–343 K. For this fuel blend, the two peaks in the emission spectra are most pronounced, even at very low temperatures. The absorption for Jet A-1 H50 shows a decrease of 9% within 375–900 nm.

A direct comparison of the investigated fuels is shown in Figure 5. The intensities of the spectra are integrated over the wavelength range investigated and normalized to the highest intensity. Between 293 K and 283 K, the change of intensity is lower than 9% for all investigated fuels. Jet A-1 shows the highest change in intensity with 8.9%. This temperature range is relevant for spray studies at moderate temperatures. For example, during the injection at low liquid fuel and ambient temperatures of 293 K, a minimal change of the droplet temperature and thus the fluorescence signal intensity would occur, as the evaporation rate is low for these fuels. In a previous study, a simulation of a pure ethanol spray at 293 K showed a maximum droplet cooling of 11 K [2]. However, the investigated fuel blends would show a decreased evaporation cooling due to the lower evaporation enthalpy (see Table 1) compared to pure ethanol. Similar results had been obtained for the gasoline-surrogate fuel n-hexane (with lower boiling point as ethanol [341 K versus 351 K] but much lower evaporation enthalpy) in ref. [60]. There, the authors studied freely falling droplets (initial diameter of 220 µm) at initial temperatures of 303 K or 343 K in air at 298 K and observed droplet temperature variations of 10–15 K as well. This means that the low temperature sensitivity of the fluorescence signal at moderate ambient temperatures (283–313 K) qualifies the investigated fuel-nile-red-mixtures as suitable for LIF/Mie droplet sizing. These moderate operating conditions (representing cold start conditions) are suitable for analysis of the spray formation of DISI engines in terms of planar droplet sizing. For conditions with larger temperature variations, a determination and correction of the temperature (e.g., by using a two-color LIF approach) is required to compensate these effects and related errors.

### 4.3. Fuel-Dependent Absorption and Emission Spectra

**Figure 6:**
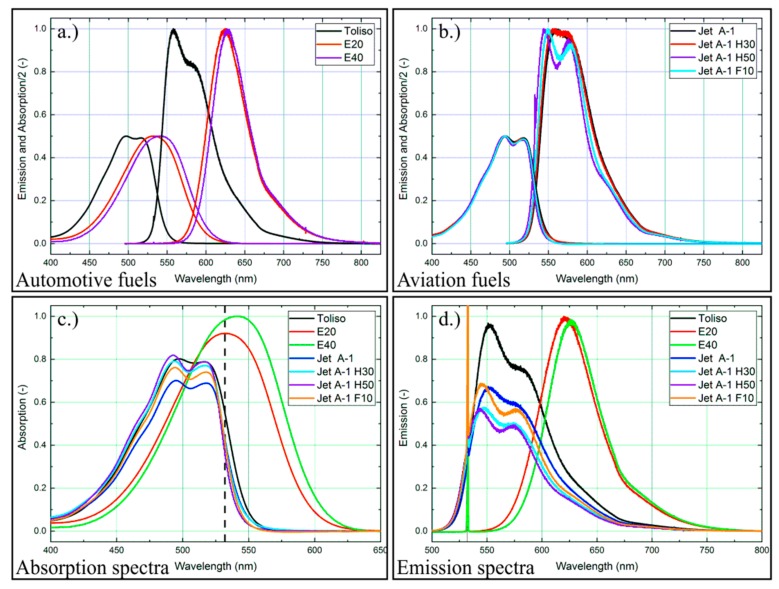
Normalized absorption and emission spectra for nile red (9.375 mg/L) for various automotive (**a**) and aviation (**b**) fuel mixtures. (Please note that the absorption signal is divided by a factor of two for clarity). Normalized absorption spectra (**c**) of the investigated fuel-dye mixtures; all spectra are normalized to the maximum absorption of E40; normalized emission spectra (**d**) of the investigated fuel-dye mixtures; all spectra are normalized to the maximum signal of E20, 293 K, 0.1 MPa.

In this section, the absorption and emission spectra of the investigated fuels are compared. This study is necessary as the fuel mixture in the droplet may vary due to preferential evaporation of certain components, which is also relevant for, as an example, ethanol-gasoline blends [39,41,61,62,63,64]. This would change the absorption and fluorescence behavior of the droplet during evaporation as well, which is another potential error when the LIF-signal is quantified, i.e., for droplet sizing based on the LIF/Mie ratio. The absorption and emission signals of the investigated fuel mixtures (nile red concentration: 9.38 mg/L) at 293 K, 0.1 MPa, are shown in Figure 6a–d. First, the spectral data of the automotive fuel mixtures are discussed (Figure 6a). As mentioned above, an ethanol admixture of 20–40% to Toliso leads to a spectral shift of about 25 nm for the absorption and 50 nm for the fluorescence signal towards higher wavelengths. The spectral red-shift of the absorption and emission signals increase slightly with a higher ethanol admixture. The ethanol blending reduces the double peak of the absorption and emission spectra to one remaining single peak. Assuming a preferential evaporation of ethanol (with remaining isooctane and toluene in the droplet, which is probable for such mixtures [41,64]), a variation in absolute signal intensity might result. This signal intensity is, however, just 1% higher for E20 in comparison to Toliso at a constant temperature and considering the complete spectral range (Figure 6d). However, at the same time, this spectral shift could be utilized to determine the ethanol content of the mixture. 

Absorption (Figure 6c) is increased for the addition of ethanol to Toliso; however, the fluorescence signal does not increase accordingly. This indicates that these large amounts of ethanol may quench the fluorescence of nile red, which was also found for different solvents in ref. [37]. Furthermore, this quenching effect is similar to the findings for the tracer TEA dissolved in ethanol containing isooctane as reported in ref. [22].

The spectral data of the investigated aviation fuel mixtures are discussed in the subsequent section. The spectral shifts between the kerosene-biofuel mixtures are much smaller compared to the Toliso-ethanol blends. In general, an admixture of the biofuel components to Jet A-1 leads to a small spectral shift to lower wavelengths in comparison to the base fuel. Jet A-1 H30 shows only a marginal spectral shift of 1–2 nm in the absorption and emission spectra, while Jet A-1 H50 shows a spectral shift of about 4 nm for the absorption and 10 nm for the fluorescence signal in comparison to Jet A-1. An admixture of 10 vol% farnesane to Jet A-1 leads to a spectral shift of about 2 nm for the absorption and 5 nm for the fluorescence signal. The biofuel admixture to Jet A-1 leads to a distinct double peak of the fluorescence signal in comparison to the weak double peak of Jet A-1. The absorption spectra show the distinct double peak for all investigated aviation fuel mixtures as well. The absolute fluorescence intensities exhibit a signal reduction with the addition of biofuels to Jet A-1, while the signal drop is maximal for Jet A-1 H50 (19%, see Figure 6d), although the absorption increase (see Figure 6c) is almost not affected at 532 nm for all Jet A-1 mixtures with biofuels. This also indicates that the fluorescence may be slightly quenched by the added biofuels, similar to the effect of ethanol as discussed above.

### 4.4. Photo-Dissociation

**Figure 7:**
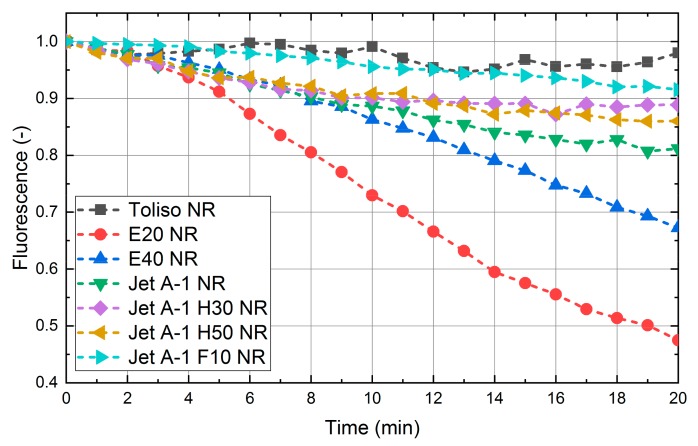
Photo-bleaching effect of the investigated fuel-dye mixtures at 293 K, 0.1 MPa. All standard deviations are <0.5% and are smaller than the symbols.

Finally, the effect of photo-dissociation, which leads to a reduction of the LIF-signal with time for continuous illumination at constant laser fluence, is discussed. For the measurements, the dye-fuel mixtures were illuminated for 20 minutes. The LIF-spectra were measured every 60 seconds, and the individual spectral fluorescence intensities were summed up (see Figure 7). The investigation of the LIF signal of the various fuels shows that only Toliso exhibits no significant change of the LIF-signal with time, while the other fuel mixtures show a more or less distinct reduction. The largest signal reduction was found for E20 and E40, while Jet A-1 and its blends only exhibit a moderate reduction. The faster photo-bleaching of E20 and E40 can be explained by the larger absorption at 532 nm compared to the other fuel mixtures. These results indicate that photo-bleaching has to be taken into account for spectral measurements within a cuvette. For spray investigations, the photo-dissociation effect is negligible, since the fuel spray is only illuminated once and the fuel is not reused, which is the case in spray studies conducted in conventional constant volume chambers [2]. 

## 5. Conclusions and Future Work

The suitability of nile red as a fluorescence dye for spectroscopic investigations was investigated for the gasoline surrogate fuel Toliso and for Jet A-1 as well as for blends with various biofuels. First, the fluorescence behavior was studied for nile red dissolved in the two base fuels, Toliso and Jet A-1, for various dye concentrations. The emission signals showed a linear trend with increasing dye concentration. Second, the temperature effect on spectral absorption and emission of nile red was investigated in a specially designed test cell. An ethanol admixture to Toliso leads to a spectral shift of about 25 nm for the absorption and 50 nm for the fluorescence band towards higher wavelengths. The absorption and emission bands are shifted with increasing temperature towards lower wavelengths for all fuels. While the absorption decreases for all fuels with higher temperatures, the fluorescence showed the same behavior, except for E20. Here, the fluorescence increased with higher temperatures. Jet A-1 and its blends with HEFA and farnesane did not exhibit distinct variations in spectral absorption and emission, but these blends showed a more pronounced temperature dependence compared to the Toliso-ethanol-blends. 

The long-time investigation of the LIF signal of the various fuels revealed that only Toliso shows no significant photo-dissociation. All other fuel mixtures exhibit a more or less distinct decay in the fluorescence signal with time. E20 and E40 show the highest signal reduction, while Jet A-1 and its blends are characterized by a moderate reduction. However, for spray studies, the photo-bleaching is not relevant, and errors may be introduced, e.g., during characterization and calibration in test cells or cuvettes, in which no exchange of the dye with time is conducted.

In summary, all investigated fuel-tracer-mixtures are suitable for planar droplet sizing using the LIF/Mie ratio in combination with nile red at moderate temperatures and low evaporation cooling rates. At elevated temperatures, which is associated with higher droplet heating or evaporative cooling, the jet fuels show a higher uncertainty in comparison to Toliso-blends. In the latter case, a temperature correction, e.g., by using a two-color LIF concept, could be realized in order to minimize these sources of uncertainty.

To fully understand the fuel- and temperature-dependent effects on the fluorescence of nile red, further fundamental characterization of the fuel-tracer mixtures has to be done. In particular, the fuel-and temperature-dependent measurement of the absorption cross-section and the fluorescence quantum yield would be necessary. However, these measurements are far beyond the focus of the present paper and will be addressed in future work by the authors. The investigated LIF concept will be applied in monodisperse droplets and sprays, for which an in-depth calibration will be conducted in future.

## Figures and Tables

**Table 1:** Physical and chemical properties of the investigated fuels, 0.1 MPa [4,45,46,47,48,49,50,51,52,53,54,55,56].

Property	Unit	isooctane	toluene	ethanol	Jet A-1	HEFA	Farnesane
H/C-ratio/O/C-ratio	-	2.25/-	1.14/-	3/0.5	1.92/-	variable	2.13/-
Boiling point	K	372	383	351	478-573	478-573	472
Density @ 293 K, 0.1 MPa	g/cm³	0.72	0.74	0.79	0.79	0.78 (@288 K)	0.77
Dynamic viscosity @ 0.1 MPa, 298 K	mPa s	0.47	0.59	1.10	1.33 (@293K)	3.90 (@253K)	2.72 (@293K)
Surface tension @ 293 K	N/m	0.019	0.029	0.022	0.027	-	0.025
Heat of vaporization @ 293 K	kJ/kg	297	364	904	300-375	-	219.8
Stoichiometric air-fuel ratio	kg/kg	15.2	13.4	9	~15	~15.3	14.9
Lower Heating Value	MJ/kg	44.3	40.6	26.8	43.45	43.7	43.6
